# Dietary Intake and Eating Behaviours of Obese New Zealand Children and Adolescents Enrolled in a Community-Based Intervention Programme

**DOI:** 10.1371/journal.pone.0166996

**Published:** 2016-11-23

**Authors:** Yvonne C. Anderson, Lisa E. Wynter, Michelle S. Butler, Cameron C. Grant, Joanna M. Stewart, Tami L. Cave, Cervantée E. K. Wild, José G. B. Derraik, Wayne S. Cutfield, Paul L. Hofman

**Affiliations:** 1 Department of Paediatrics, Taranaki District Health Board, New Plymouth, New Zealand; 2 Liggins Institute, University of Auckland, Auckland, New Zealand; 3 Department of Paediatrics, University of Auckland, Auckland, New Zealand; 4 Starship Children’s Health, Auckland District Health Board, Auckland, New Zealand; 5 Centre for Longitudinal Research - He Ara ki Mua, University of Auckland, Auckland, New Zealand; 6 Department of Epidemiology and Biostatistics, University of Auckland, Auckland, New Zealand; Weill Cornell Medical College in Qatar, QATAR

## Abstract

**Objectives:**

The aim of this study was to describe dietary intake and eating behaviours of obese children and adolescents, and also to determine how these differ in Indigenous versus non-Indigenous children at enrolment in an obesity programme.

**Methods:**

Baseline dietary intake and eating behaviour records were assessed from those enrolled in a clinical unblinded randomised controlled trial of a multi-disciplinary intervention. The setting was a community-based obesity programme in Taranaki, New Zealand. Children or adolescents who were enrolled from January 2012 to August 2014, with a BMI ≥98^th^ percentile or >91^st^ centile with weight-related comorbidities were eligible.

**Results:**

239 participants (45% Māori, 45% NZ Europeans, 10% other ethnicities), aged 5–17 years were assessed. Two-thirds of participants experienced hyperphagia and half were not satiated after a meal. Comfort eating was reported by 62% of participants, and daily energy intake was above the recommended guidelines for 54%. Fruit and vegetable intake was suboptimal compared with the recommended 5 servings per day (mean 3.5 [SD = 1.9] servings per day), and the mean weekly breakfasts were less than the national average (5.9 vs 6.5; p<0.0001). Median sweet drink intake amongst Māori was twice that of NZ Europeans (250 vs 125 ml per day; p = 0.0002).

**Conclusions:**

There was a concerning prevalence of abnormal eating behaviours and significant differences in dietary intake between obese participants and their national counterparts. Ethnic differences between Indigenous and non-Indigenous participants were also present, especially in relation to sweet drink consumption. Eating behaviours, especially sweet drink consumption and fruit/vegetable intake need to be addressed.

## Introduction

Childhood obesity is an issue of global importance. The World Health Organization has multiple recommendations in order to curb the obesogenic environment, and these include improving intake of healthy foods and physical activity levels [1,2]. Influencing the food environment is complex, requiring a co-ordinated approach across all of society [2]. International focus has recently been placed on limiting free sugar intake in order to help prevent unhealthy weight gain, with the World Health Organization recommending a reduction of free sugars to <10% of total energy intake, both in adults and children [3].

New Zealand has the 3^rd^ highest prevalence of overweight and obesity in children and adults among OECD countries [4]. Māori (New Zealand’s Indigenous population) aged 2 to 14 years of age are 1.6 times more likely to be obese compared with non-Māori counterparts, and Pacific children 3.6 times more likely than non-Pacific children [5]. Despite this, there have been few in-depth surveys of child nutrition in New Zealand [5–7].

Differences in eating patterns have been noted between ethnic groups; Māori and Pacific children were more likely to have a less healthy diet when compared with New Zealand European (NZE) children. The median daily energy intake for Māori children was higher than NZE and Pacific children, and Māori had the highest intake of sucrose [6]. Factors that were independently associated with the likelihood of eating breakfast at home every day were ethnicity (Māori and Pacific children less likely) and socioeconomic status (children living in more deprived households less likely) [5].

Eating behaviours play an important role in healthy eating. Studies have shown that a variety of eating behaviour traits are associated with weight in children [8]. Satiety responsiveness/slowness in eating (combined to form a single subscale) and food fussiness in children were negatively associated with body weight, whilst food responsiveness, enjoyment of food, emotional overeating and desire to drink were positively associated with weight [8]. Obese children have been noted to eat faster than their non-obese counterparts [9]. In a New Zealand study, a healthy diet (defined by specific items assessing healthy eating behaviours) was associated with better emotional health in 12- to 17-year old children [10].

When tackling childhood obesity, a multi-disciplinary approach is recommended, working with families to address food habits, physical activity and (lifestyle) behaviour [11]. Our primary objective in reporting the data in this manuscript was to describe the eating behaviours of the children and adolescents referred to the ‘Whānau Pakari’ programme, and how these compared with regional and national cohort data. Our secondary objective was to determine whether eating behaviours differed between the Indigenous (Māori) and non-Indigenous children and adolescents enrolled in the programme.

## Methods

Recruitment was from participants referred to a community-based, unblinded randomised controlled trial of a multi-disciplinary obesity intervention programme, based in Taranaki, a region of New Zealand (population of 23,139 children aged 0–15 years, of which 81% identify as NZE, 28% as Māori, and 1% as other ethnicity) [12]. Details of the study methods including sample size and power calculations have been previously published [13]. Recruitment period was between January 2012 and August 2014. Eligibility was defined by residence in Taranaki, aged from 5 to 16 years, and being either obese or overweight as defined by a modification of the United Kingdom Cole definitions of BMI ≥98^th^ centile, and >91^st^ centile with weight-related comorbidities respectively [14]. Participants were identified as obese within the community or on hospital visits, and referred by health professionals, Māori health workers, school counsellors, and by family members/self-referrals.

This study was conducted according to the guidelines laid down in the Declaration of Helsinki and all procedures involving human subjects/patients were approved by the New Zealand Health and Disability Ethics Committee (CEN/11/09/054). Written and verbal informed consent was obtained from all the participant’s parents or guardians, using an information and consent form approved by the Ethics Committee. In addition, verbal consent was also obtained from all participants, as well as written consent as appropriate for their age. This consent form was stored in participant’s clinical records. Trial registration was with the Australian New Zealand Clinical Trials Registry (ANZCTR: 12611000862943, https://www.anzctr.org.au/Trial/Registration/TrialReview.aspx?id=343300&isReview=true).

### Data Collection

The Healthy Lifestyles Coordinator undertook a baseline assessment at each participant’s home. Data collected included weight-related assessments (including measured weight and height), a focussed medical, dietary, physical and psychological review (including measuring heights and weights) [13], as well as fasting blood samples. Baseline comorbidity data (including metabolic data) have been reported [15].

Dietary assessment included a dietetic history from the accompanying adult, a 24-hour food recall, and administration of the validated Children’s Dietary Questionnaire, modified for use in New Zealand [16]. The 24-hour recall was completed with the child’s caregiver or with both the caregiver and the study participant if aged >11 years.

We compared the data from our participants aged 5 to 14 years with that collected at the 2012/2013 New Zealand Health Survey (NZHS), making these comparisons both with the NZHS national data and the dataset limited to study participants living in the Taranaki region. The NZHS included a random sample of predominantly non-obese children aged 5 to 14 years (n = 4,699) with data collected from their caregivers, allowing comparisons with our dataset for the number of breakfasts eaten per week, the number of take away meals per week, and number of fruit servings per day [17].

### Measures

BMI percentile and BMI standard deviation score (SDS) were calculated using UK Cole normative data [18] with the KIGS auxology software (Pfizer Endocrine Care TM). Socioeconomic deprivation was measured at the household level using the New Zealand Deprivation Index 2006, and socioeconomic status was coded as decile of household deprivation [19]. This area level deprivation index, which is derived from national census data on nine socioeconomic characteristics, is a well-validated measure of small area socioeconomic deprivation in New Zealand. Age was coded as age at assessment.

FoodWorks 7 Professional Edition (published by Xyris Software Australia Pty Ltd, Brisbane 2012) was used to determine the dietary composition of the participant’s 24-hour recall. This programme is derived from the Australia and New Zealand Nutrient Reference Values [20]. Energy requirements, as based on the participant’s age, gender, and ideal weight for height percentile were calculated to determine if energy intake was above the participant’s recommended daily intake.

The number of serves per day from each of the four main food groups (vegetables and fruit; breads and cereals; milk and milk products; and lean meat, poultry, seafood, eggs, legumes, nuts and seeds) were determined from the dietary recall. In New Zealand, the recommendations for daily food group consumption for children aged 5 to 14 years include 3 servings of vegetables and at least 2 servings of fruit; 5 servings of breads and cereals; 2 to 3 servings of milk and milk products and 2 servings of lean meat, poultry, seafood, eggs, legumes, nuts and seeds [21]. Adequate intake across the four food groups was then determined.

### Data Analyses

To investigate variables associated with categorical outcomes of interest including hyperphagia, lack of satiety after food, night waking for food, comfort eating, and energy intake above the recommended daily intake, binary logistic regressions, with models including variables that described age, sex, ethnicity, and socioeconomic deprivation (as well as BMI SDS where appropriate) were run. For continuous outcomes: number of breakfasts consumed per week; total energy intake; percentage of energy intake from carbohydrate, fat, and protein; number of daily servings of fruit, vegetables, and snack foods, general linear models were run including the same explanatory variables as above. A two-sample t-test was used to examine differences between Whānau Pakari and New Zealand Health Survey data as well as to look at differences in age and BMI between Māori and NZE participants. Differences between the latter ethnic groups were also examined using the following methods: a chi-squared test for sex ratio; a Kruskal-Wallis test for specific outcomes (i.e. socioeconomic status; daily milk, water, and sweet drink consumption); and a general linear model or a binary logistic regression (both including age, sex, and socioeconomic status as predictors) for the remaining continuous and binary outcomes, respectively. Analyses were performed in Minitab (v.16, Pennsylvania State University, State College, PA, USA) and SAS v.9.4 (SAS Institute, Cary, NC, USA). All statistical tests were two-tailed with a significance level maintained at p<0.05.

## Results

Enrolled participants (n = 239) had a mean age of 10.7 years (SD = 3.2, range 5–17 years), 52% of whom were females. Participants were predominantly of Māori (45%) or NZE (45%) ethnicity, with the remainder being of Pacific (3%), Asian (3%), or other ethnicities (4%). Twenty-nine percent resided in households that were among the most deprived quintile of New Zealand households (compared with fifteen percent of the total population of Taranaki) [19,22,23]. Mean BMI SDS at enrolment was 3.1 (SD = 0.60, range 1.52–5.34).

### Dietary History and Intake

Table 1 summarises proxy and self-reports of eating behaviour of participants. Two-thirds of the children were reported to experience hyperphagia (excessive hunger and eating large amounts of food), and half were not satiated after a meal (Table 1).

**Table 1:** Reported eating behaviours of the 239 study participants aged 5 to 17 years who were either obese or overweight. Data are n (%), or mean (standard deviation) where stated.

Hyperphagia^a^	160 (67%)
Night waking for food	21 (9%)
Not satiated after food	122 (51%)
Comfort eater	147 (62%)
All lunches prepared at home	100 (42%)
All dinners prepared at home	74 (31%)
Breakfasts eaten per week (n) †	5.6 (2.3)

^†^ Data are mean (SD).

^a^ Responded "yes" to the question “Does your child/teen seem excessively hungry and eat very large amounts of food?”

For every 1 SDS increase in BMI, the odds of experiencing hyperphagia were over 2.5 times greater (Table 2). The odds of experiencing lack of satiety after eating were twice as high in females compared with males (Table 2). In addition, the number of breakfasts eaten per week decreased with increasing age (Table 2).

**Table 2:** The association between the predictor variables and eating habits among Whānau Pakari participants.

Predictors	Hyperphagia		Lack of satiety after food		Breakfasts per week (n)	
	OR (95% CI)	*p*	OR (95% CI)	*p*	β (95% CI)	*p*
BMI SDS	2.64 (1.44, 4.82)	0.002	0.86 (0.52, 1.44)	0.57	-0.38 (-0.87, 0.10)	0.12
Sex (female vs male)	0.71 (0.37, 1.34)	0.29	1.96 (1.09, 3.53)	0.024	-0.49 (-1.06, 0.07)	0.08
Socioeconomic status (NZDep2013)	0.99 (0.87, 1.12)	0.88	1.10 (0.98, 1.24)	0.12	-0.08 (-0.19, 0.03)	0.16
Age at assessment (years)	1.03 (0.92, 1.17)	0.60	1.06 (0.95, 1.18)	0.32	-0.20 (-0.28, -0.11)	<0.0001
Ethnicity (Māori vs NZ European)	0.83 (0.42, 1.65)	0.92	1.03 (0.55, 1.94)	0.43	-0.61 (-1.21, 0.00)	0.05

CI, confidence interval. Data for hyperphagia and lack of satiety were analysed using binary logistic regression models, while data for breakfasts eaten were analysed using a general linear regression model; all variables included as predictors are listed in the table.

Table 3 describes proxy and self-reported dietary intake of participants. Over half (54%) of the children had an energy intake that exceeded their recommended daily intake, based on age and gender. There was no association between BMI SDS and total energy intake, irrespective of adjustment for confounders (Table 4).

**Table 3:** Reported dietary intake of participants (n = 238) aged 5 to 17 years who were either obese or overweight. Data are means (standard deviations), medians (IQR; ranges), or n (%).

**Energy source (% total energy intake)**	Carbohydrate †	46.0 (10.0)
	Protein †	16.0 (6.0)
	Fat †	37.0 (9.0)
**Energy intake per day (kcal)**	Total energy intake †	2013 (731.0)
	Energy intake above calculated RDI	127 (54%)
	Adequate intake across all four food groups^a^	6 (3%)
**Servings per day (n)**	Fruit †	1.8 (1.3)
	Vegetables †	1.7 (1.0)
	Combined fruit and vegetables †	3.5 (1.9)
	Snack foods^b^ †	1.9 (1.1)
**Fluid consumption (ml/day)**	Water ‡	800 (500; 0–5,000)
	Milk ‡	250 (200; 0–1,000)
	Sweet drink^c^ ‡	250 (450; 0–2,000)
**Fast food/take aways per week (n)** †		1.2 (1.0)

RDI, recommended daily intake [20].

^†^ Data are means (SD).

^‡^ Data are medians (interquartile range; range).

^a^ Defined as meeting recommended number of servings of the four main food groups [21].

^b^ Includes crisps (potato chips), sweets, cake/biscuits, and muesli bars.

^c^ Includes powdered drinks, cordial, fruit juice, energy drinks, and carbonated sweet drinks.

**Table 4:** The association between the predictor variables and total daily energy intake (kcal) among Whānau Pakari participants.

Predictors	Total energy intake (kcal)	
	β (95% CI)	*p*-value
BMI SDS	47 (-108, 202)	0.55
Sex (female vs male)	-322 (-502, -143)	0.0005
Socioeconomic status (NZDep2013)	29 (-6, 64)	0.10
Age at assessment (years)	50 (23, 78)	0.0004
Ethnicity (Māori vs NZ European)	83 (-110, 277)	0.40

CI,confidence interval. Data were analysed using a general linear regression model.

Comparative figures for national intake in New Zealand children and young people (aged 5–18 years) based on 24-hour recall proxy and self-report in past nutrition surveys show 48–54% of energy source is carbohydrate, with fat making up 33–35% of total energy, and protein 14–16% of total energy, which is similar to what we found in our cohort [6,21].

Servings of fruit and vegetables in this cohort were both below the recommended intake of ≥2 fruit (mean 1.8 [SD = 1.3]) servings per day and ≥3 vegetable (mean 1.7 [SD = 1.0]) servings per day [21]. Only 3% of participants met the New Zealand recommendations for number of servings from the four main food groups.

### Comparisons with New Zealand Health Survey

Compared to national NZHS data, participants ate fewer breakfasts per week (mean 5.9 vs 6.5, p<0.0001) and more servings per week of fast food/take aways (mean 1.3 vs 1.0, p = 0.006) per week (Table 5) [17].

**Table 5:** Eating behaviours among children aged 5 to 14 years from Whānau Pakari (n = 193) and the New Zealand Health Survey (NZHS) 2012–2013 [17]. Whānau Pakari data are compared to national (n = 2,724) and regional (Taranaki; n = 128) data.

	Whānau Pakari	NZHS—National		NZHS—Taranaki	
	Mean (SD)	Mean (SD)	p-value	Mean (SD)	p-value
Breakfast eaten per week (n)	5.9 (2.2)	6.5 (1.7)	<0.0001	6.3 (2.1)	0.11
Servings of fruit per day (n)	1.9 (1.3)	2.2 (1.5)	0.007	2.0 (1.7)	0.55
Fast food/take aways per week (n)	1.3 (0.9)	1.0 (1.5)	0.006	1.4 (1.5)	0.46

Data were analysed using two-sample t-tests, separately comparing national and Taranaki data with Whānau Pakari data. Thus, p-values provided are for comparisons with Whānau Pakari.

During this time period (2011–2013), Taranaki had the second highest regional prevalence of obesity in the 2- to 14-year age group in the NZHS (22%), and double the national childhood obesity rate (11%) [17].

### Ethnic Comparisons within the Whānau Pakari Cohort

Due to the low representation of Pacific peoples, Asian and other ethnicities, ethnic comparisons were only made between Māori and NZE.

Māori had higher BMI SDS at entry compared with NZE (mean 3.20 [SD = 0.66] vs 2.99, respectively; Table 6). Māori participants consumed a greater median volume of sweet drinks than NZE (250 vs 125 ml, respectively; Table 6).

**Table 6:** Demographic characteristics and study outcomes in Māori and New Zealand European participants from Whānau Pakari.

		Māori	NZ Europeans	*p*-value
N		109	108	
Demography	BMI SDS † §	3.20 (0.66)	2.99 (0.51)	0.01
	Sex ratio (females) ¥	57%	50%	0.31
	Socioeconomic status (NZDep2006) ‡ ¶	8 [3; 1–10]	6 [4; 1–10]	<0.001
	Age at assessment (years) † §	10.3 (3.2)	10.8 (3.4)	0.25
Eating behaviours	Breakfasts eaten (n) † *	5.3 (2.4)	5.9 (2.2)	0.01
	Hyperphagia #	68%	67%	0.89
	Night waking for food #	9%	8%	0.59
	Lack of satiety after meals #	46%	50%	0.89
	Comfort eating #	59%	64%	0.53
Dietary intake	Energy from carbohydrate (% total) † *	45 (9)	45 (11)	0.60
	Energy from fat (% total) † *	38 (8)	37 (10)	0.48
	Energy from protein (% total) † *	15 (5)	17 (6)	0.05
	Total energy intake (kcal) † *	2031(815)	1946(632)	0.42
	Energy intake above RDI #	54%	52%	0.56
	Fruit servings per day (n) † *	3.5 (2.0)	3.6 (1.8)	0.43
	Vegetable servings per day (n) † *	1.6 (1.1)	1.9 (1.0)	0.06
	Snack servings per day (n) † *	2.0 (1.1)	1.9 (1.0)	0.34
Fluid intake	Water (ml/day) ‡ ¶	800 [750; 0–5000]	900 [750; 0–3000]	0.62
	Milk (ml/day) ‡ ¶	250 [143; 0–1000]	250 [250; 0–750]	0.80
	Sweet drinks (ml/day) ‡ ¶	250 [410; 0–2000]	125 [250; 0–1000]	<0.001

^†^ Data are means (SD).

^‡^ Data are medians (interquartile range; range).

* Data analysed using a general linear regression model, including ethnicity (Māori and NZ European), socioeconomic status, age at assessment, and sex as explanatory variables.

^¥^ Data analysed using a chi-square test.

^§^ Data analysed with a two-sample t-test.

^¶^ Data analysed with a Kruskal-Wallis test.

^**#**^ Data analysed using a binary logistic regression model, including ethnicity (Māori and NZ European), socioeconomic status, age at assessment, and sex as explanatory variables.

Māori ate fewer breakfasts compared with NZE (average per week 5.3 vs 5.9; Table 6). However, Māori and NZE had similar rates of hyperphagia, night waking for food, lack of satiety after meals, and comfort eating (Table 6).

Māori and NZE children and adolescents had similar proportions of their energy intake from carbohydrate and fat, but Māori tended to have a smaller proportion of energy intake from protein (15% vs 17%; Table 6). The proportion of participants with energy intake above the calculated recommended daily intake (RDI) was similar in Māori (54%) and NZE (52%, p = 0.89).

Māori and NZE had similar average number of daily servings of fruit, vegetables, and snack foods, and a similar intake in ml per day of water and milk (Table 6).

## Discussion

Our findings show a concerning prevalence of abnormal eating behaviours in obese participants, with noteworthy differences in dietary intake compared to their predominantly non-obese national counterparts. Ethnic differences between Indigenous and non-Indigenous participants were also present. However, we expected the differences in our participants to be greater, and believe that three factors may have contributed to this. The first was the inability to undertake food diaries where prospective data could be collected; whilst we attempted these, participants and their families did not reliably complete them and we were left undertaking 24-hour recall. Secondly, intake is likely to be under-reported in our cohort. Finally, whilst there is minimal difference in caloric intake between our participants and national population-based surveys, this does not reflect the quality of calories such as fat and carbohydrate consumed in the data collected.

We saw abnormalities in the psychological aspects of eating behaviours in obese children and adolescents. Excessive hunger, eating large amounts of food, comfort eating, and lack of satiety were commonly reported in this cohort. Emotional eating behaviour and shorter duration of eating have been found to be associated with increased weight in previous studies [8]. Clinicians dealing with obese children need to be aware of the psychological dimensions of dietary behaviours, in order to tailor any intervention effectively [24].

A key difference we observed between Indigenous versus non-Indigenous participants was that Māori obese children and adolescents drank twice the volume of sweet drinks of their NZE counterparts. With a median sweet drink consumption across the whole cohort of 250 ml per day, the obese children and adolescents in this cohort are gaining an additional load of 100kcal per day from sweet drinks. Whilst this may not appear to be a large amount when compared with total energy intake, extra energy intake of 120kcal per day is estimated to lead to a 50-kg increase in body mass over 10 years [25]. A greater consumption of sugar sweetened beverages is associated with obesity [26]. Importantly, this includes not only carbonated sweet drinks, but also powdered fruit drink which were the most popular non-dairy beverage among New Zealand children in the National Children’s survey in 2002 [6]. We were unable to directly compare sweet drink consumption with national data as the NZHS asked only about carbonated (“fizzy or soft”) drinks rather than all sweet drinks.

Replacement of sugar sweetened beverages with non-caloric artificially sweetened beverages has been shown to reduce weight gain and fat accumulation in normal weight children [27]. However, diet drinks are not recommended in children as they can cause tooth erosion due to their acidity, and maintain a preference for sweetness [21]. Therefore, a population-based move towards artificially sweetened beverages is controversial. Emerging evidence, mostly in animals, shows artificial sweeteners can alter sweet preferences, interfere with learning of the relationship between sweet tastes and delivery of calories, and can alter the composition of gut microbiota, negatively affecting metabolic regulation [28].

This study demonstrated alterations to dietary composition that are utilised as overall indicators of a healthy diet, such as number of servings of fruit and eating breakfast. These were below national guidelines and intake in national surveys. Fruit and vegetables contribute (in part) to satiety, provide many nutrients, and are also a source of dietary fibre [21]. Ways to improve fruit and vegetable intake in obese children and adolescents need to be considered, and given the diversity in fruit and vegetable intake in different ethnic groups of children in New Zealand [6], a more culturally responsive approach may need to be adopted than conventional dietary guidelines can provide [29]. Some countries, for example Brazil, are now using guidelines as an opportunity to promote the health and well-being of people, an avenue which may garner broad appeal, especially across multiple ethnic groups [29]. Availability and accessibility of healthy food varies [30], as does food security between families based upon their household deprivation and ethnicity. These factors need to be taken into account in any attempt to address eating behaviours in the obese child and adolescent population [31].

Children who miss breakfast are less likely to meet recommendations for fruit and vegetable consumption, and are more likely to have a raised BMI [32]. Participants in our cohort ate fewer breakfasts per week compared with national data. As the NZHS asked how many breakfasts were eaten at home in the past 7 days, whereas we asked the number of breakfasts eaten in the past 7 days, the differences reported here between our cohort and the national data are likely to be an underestimate. Our findings that older children are more likely to miss breakfast were consistent with previous research findings [32,33]. Of note, Māori children and adolescents were more likely to miss breakfast compared with NZE. This is important, given that “breakfast skippers” are more likely to have inadequate dietary intake [33].

A strength of this study was our high participation rate from Māori, allowing for robust comparison between Indigenous and non-Indigenous participants. The key limitation of our study (and of many dietary studies) is the use of 24-hour food recall with proxy report and self-report. Self-report has been questioned due to many notable inaccuracies, and some are now arguing that this method is so unreliable that it should not be utilised at all, given implications for policy on findings published in the literature [34]. There are many factors that affect accuracy of recall of dietary intake in children, including BMI and retention interval [35]. Given our data is likely to represent under-reporting of intake, it is probable that the dietary intake of many of these children is worse than we have reported [36]. Some data points were not directly comparable with the NZHS, which limited the ability to compare our cohort directly with national data; an example being vegetable servings per day (NZHS included potato) [17].

In this cohort, dietary composition as reported by 24-hour recall was not dissimilar to that described in previous childhood population studies in New Zealand [6,21]. However, over half of the children and adolescents we studied had an energy intake greater than their RDI. Interestingly, there was no association between energy intake and BMI SDS, although it is important to note the high entry BMI SDS of participants. Firm conclusions with regard to portion size cannot be made, given the known challenges of dietary under-reporting, especially in obese individuals [36]. It is likely that quality of carbohydrate, fat and other dietary sources is also an important factor in this cohort. This highlights the need for observational methods (such as wearable digital cameras) for gathering dietary information in such a cohort to improve accuracy of reporting in future research.

In conclusion, this study highlights there are specific eating behaviours that need targeting for obese children, adolescents, and their families, and approaches that appeal to all ethnic groups need to be considered. Future research utilising observational methods to determine more accurate food intake is necessary.

## References

[1] World Health Organization. Interim Report of the Commission on Ending Childhood Obesity. 2015. Available from: http://www.who.int/end-childhood-obesity/commission-ending-childhood-obesity-interim-report.pdf

[2] World Health Organization. Report of the Commission on Ending Childhood Obesity. 2016. Available from: http://apps.who.int/iris/bitstream/10665/204176/1/9789241510066_eng.pdf?ua=1

[3] World Health Organization. Guideline: Sugars intake for adults and children. 2015. Available from: http://apps.who.int/iris/bitstream/10665/149782/1/9789241549028_eng.pdf?ua=1 25905159

[4] Organisation for Economic Cooperation and Development (OECD). Obesity Update 2014. 2014. Available from: http://www.oecd.org/els/health-systems/Obesity-Update-2014.pdf

[5] Ministry of Health, New Zealand. Annual Update of Key Results 2014/2015: New Zealand Health Survey. 2015. Available from: http://www.health.govt.nz/system/files/documents/publications/annual-update-key-results-2014-15-nzhs-dec15-1.pdf

[6] Ministry of Health, New Zealand. NZ Food NZ Children: Key Results of the 2002 National Children's Nutrition Survey. 2003. Available from: https://www.health.govt.nz/system/files/documents/publications/nzfoodnzchildren.pdf

[7] Clinical Trials Research Unit; Synovate. A National Survey of Children and Young People's Physical Activity and Dietary Behaviours in New Zealand: 2008/09—Key Findings. 2010. Available from: http://www.health.govt.nz/publication/national-survey-children-and-young-peoples-physical-activity-and-dietary-behaviours-new-zealand-2008.

[8] Webber L, Hill C, Saxton J, Van Jaarsveld CH, Wardle J. Eating behaviour and weight in children. Int J Obes (Lond). 2009;33: 21–28. 10.1038/ijo.2008.219 19002146 PMC2817450

[9] Barkeling B, Ekman S, Rossner S. Eating behaviour in obese and normal weight 11-year-old children. Int J Obes Relat Metab Disord. 1992;16: 355–360. 1319970

[10] Kulkarni AA, Swinburn BA, Utter J. Associations between diet quality and mental health in socially disadvantaged New Zealand adolescents. Eur J Clin Nutr. 2015;69: 79–83. 10.1038/ejcn.2014.130 25028085

[11] Ministry of Health, Clinical Trials Research Unit, New Zealand. Clinical Guidelines for Weight Management in New Zealand Children and Young People. 2009. Available from: www.health.govt.nz/publication/clinical-guidelines-weight-management-new-zealand-children-and-young-people.

[12] Statistics New Zealand. Census 2013: Ethnic group by age group and sex. Available from: http://nzdotstat.stats.govt.nz/wbos/Index.aspx?DataSetCode=TABLECODE8021#

[13] Anderson YC, Wynter LE, Moller KR, Cave TL, Dolan GMS, Grant CC, et al The effect of a multi-disciplinary obesity intervention compared to usual practice in those ready to make lifestyle changes: design and rationale of Whānau Pakari. BMC Obesity. 2015;2: 41 10.1186/s40608-015-0068-y 26464806 PMC4599755

[14] Cole TJ. A chart to link child centiles of body mass index, weight and height. Eur J Clin Nutr. 2002;56: 1194–1199. 10.1038/sj.ejcn.1601473 12494304

[15] Anderson YC, Wynter LE, Treves KF, Grant CC, Stewart JM, Cave TL, et al Prevalence of comorbidities in obese New Zealand children and adolescents at enrolment in a community-based obesity programme. J Paediatr Child Health. 2016 10.1111/jpc.13315 27634284

[16] Magarey A, Golley R, Spurrier N, Goodwin E, Ong F. Reliability and validity of the Children's Dietary Questionnaire: A new tool to measure children's dietary patterns. Int J Pediatr Obes. 2009;4: 257–265. 10.3109/17477160902846161 19922040

[17] Ministry of Health, New Zealand. New Zealand Health Survey: Annual Update of Key Findings 2012/13. 2013. Available from: http://www.health.govt.nz/system/files/documents/publications/new-zealand-health-survey-annual-update-2012-13-dec13-v3.pdf

[18] Freeman JV, Cole TJ, Chinn S, Jones PR, White EM, Preece MA. Cross sectional stature and weight reference curves for the UK, 1990. Arch Dis Child. 1995;73: 17–24. 7639543 10.1136/adc.73.1.17 PMC1511167

[19] University of Otago—School of Medicine and Health Science, New Zealand. New Zealand Deprivation Index 2006. 2011. Available from: https://koordinates.com/layer/1066-nz-deprivation-index-2006/

[20] National Health and Medical Research Council, Department of Health and Ageing, Australian Government; Ministry of Health, New Zealand. Nutrient Reference Values for Australia and New Zealand, including Recommended Dietary Intakes. 2006. Available from: https://www.nhmrc.gov.au/_files_nhmrc/publications/attachments/n35.pdf

[21] Ministry of Health, New Zealand. Food and Nutrition Guidelines for Healthy Children and Young People (Aged 2–18 Years) A background paper. 2012. Available from: http://www.health.govt.nz/system/files/documents/publications/food-nutrition-guidelines-healthy-children-young-people-background-paper-feb15-v2.pdf

[22] Ministry of Health. Population of Taranaki DHB. 2015. Available from: http://www.health.govt.nz/new-zealand-health-system/my-dhb/taranaki-dhb/population-taranaki-dhb

[23] Atkinson J, Salmond C, Crampton C. NZDep2013 Index of Deprivation. 2014. Available from: http://www.otago.ac.nz/wellington/otago069936.pdf

[24] Ho M, Gow M, Halim J, Chisholm K, Baur LA, Noakes M, et al Effect of a prescriptive dietary intervention on psychological dimensions of eating behavior in obese adolescents. Int J Behav Nutr Phys Act. 2013;10: 119 10.1186/1479-5868-10-119 24156290 PMC3842818

[25] Ebbeling CB, Pawlak DB, Ludwig DS. Childhood obesity: public-health crisis, common sense cure. Lancet. 2002;360: 473–482. 10.1016/S0140-6736(02)09678-2 12241736

[26] Malik VS, Pan A, Willett WC, Hu FB. Sugar-sweetened beverages and weight gain in children and adults: a systematic review and meta-analysis. Am J Clin Nutr. 2013;98: 1084–1102. 10.3945/ajcn.113.058362 23966427 PMC3778861

[27] de Ruyter JC, Olthof MR, Seidell JC, Katan MB. A trial of sugar-free or sugar-sweetened beverages and body weight in children. N Engl J Med. 2012;367: 1397–1406. 10.1056/NEJMoa1203034 22998340

[28] Swithers SE. Artificial sweeteners are not the answer to childhood obesity. Appetite. 2015;93: 85–90. 10.1016/j.appet.2015.03.027 25828597

[29] Monteiro CA, Cannon G, Moubarac JC, Martins AP, Martins CA, Garzillo J, et al Dietary guidelines to nourish humanity and the planet in the twenty-first century. A blueprint from Brazil. Public Health Nutr. 2015;18: 2311–2322. 10.1017/S1368980015002165 26205679 PMC10271430

[30] Wang J, Williams M, Rush E, Crook N, Forouhi NG, Simmons D. Mapping the availability and accessibility of healthy food in rural and urban New Zealand—Te Wai o Rona: Diabetes Prevention Strategy. Public Health Nutr. 2010;13: 1049–1055. 10.1017/S1368980009991595 19781125

[31] Rush E, Puniani N, Snowling N, Paterson J. Food security, selection, and healthy eating in a Pacific Community in Auckland, New Zealand. Asia Pac J Clin Nutr. 2007;16: 448–454. 17704026

[32] Utter J, Scragg R, Mhurchu CN, Schaaf D. At-home breakfast consumption among New Zealand children: associations with body mass index and related nutrition behaviors. J Am Diet Assoc. 2007;107: 570–576. 10.1016/j.jada.2007.01.010 17383261

[33] Rampersaud GC, Pereira MA, Girard BL, Adams J, Metzl JD. Breakfast habits, nutritional status, body weight, and academic performance in children and adolescents. J Am Diet Assoc. 2005;105: 743–760. 10.1016/j.jada.2005.02.007 15883552

[34] Dhurandhar N, Schoeller D, Brown A, Heymsfield S, Thomas D, Sørensen T, et al Energy balance measurement: when something is not better than nothing. Int J Obes. 2015;39: 1109–1113. 10.1038/ijo.2014.199 PMC4430460 25394308

[35] Sharman SJ, Skouteris H, Powell MB, Watson B. Factors related to the accuracy of self-reported dietary intake of children aged 6 to 12 years elicited with interviews: a systematic review. Journal of the Academy of Nutrition and Dietetics. 2016;116: 76–114. 10.1016/j.jand.2015.08.024 26482094

[36] Heitmann BL, Lissner L. Dietary underreporting by obese individuals—is it specific or non-specific? BMJ. 1995;311: 986–989. 7580640 10.1136/bmj.311.7011.986 PMC2550989

